# Chronic irreducible dislocation of the proximal interphalangeal joint of the fifth toe: a case report

**DOI:** 10.1186/1756-0500-7-76

**Published:** 2014-02-04

**Authors:** Tanawat Vaseenon, Chaiyarit Cheewawattanachai, Yuddhasert Sirirungruangsarn

**Affiliations:** 1 Orthopaedic Department, Faculty of Medicine, Chiang Mai University, 110 Intavaroros Road, Chiang Mai 50200, Thailand

**Keywords:** Interphalangeal joint dislocation, Irreducible, Injury, Foot, Toe

## Abstract

**Background:**

Traumatic dislocation of the interphalangeal of the fifth toe is an unusual foot injury.

**Case presentation:**

We report the case of a 47-year-old woman who sustained a minor foot injury for more than 30 years, resulting in chronic, irreducible dislocation of the proximal interphalangeal joint of the fifth toe. The affected proximal interphalangeal joint was accessed via a dorsal incision over the unstable interphalangeal joint. It was found that the interposed interphalangeal joint capsule and attenuated lateral collateral ligament were reconstructed, and it was stabilized by temporary insertion of a Kirschner wire. The affected joint was found to be stable, well-positioned and pain-free at the 12-month post-surgical check-up.

**Conclusion:**

This unusual presentation of a chronic joint dislocation responded favorably to open reduction, soft tissue reconstruction and restabilization of the affected joint. It is suggested that this approach will provide a good and functional outcome even in cases of very long-standing joint injury.

## Background

Isolated, traumatic dislocation of interphalangeal (IP) toe joint is an uncommon foot injury more usually occurring in association with other foot injuries [1]. The great toe is the most commonly affected [1-7]. Healing by closed reduction is usually successful and there is either the hallux or lesser toes’ involvement [8]. However, irreducible distal interphalangeal (DIP) or proximal interphalangeal (PIP) joint dislocation of the toes may require open surgical reduction in order to achieve joint stability and restore function [4,5,9]. Irreducible dislocation occurs when the plantar plate of the collateral joint ligament, or the flexor tendon becomes interposed between the bone ends of the involved joint [2,3,5,7].

Non-resolving, chronic IP joint dislocation affecting the PIP joint of the fifth toe is an uncommon foot injury. When it occurs, it is usually the result of abductory forces causing dorsolateral displacement of the distal segment of the fifth toe [9]. The joint dislocation seems irreducible when the associated medial collateral ligament or the flexor digitorum longus tendon is entrapped within the incongruent joint. The other reason is that the buttonhole effect between the fibers of extensor tendon and the proximal phalanx, will require surgical intervention to achieve joint stability and restore normal function [7].

It is shown that the case of chronic irreducible dislocation of the PIP joint of the fifth toe occurs and it can be resoluted by opening the reduction of the dislocation. The mid-term (a 12-month post surgery) results in the intervention are presented.

## Case presentation

**Figure 1:**
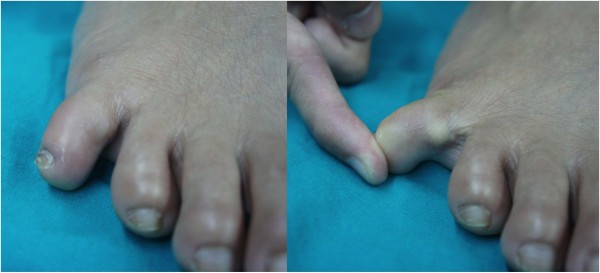
Preoperative clinical presentation of the chronic irreducible dislocation of the proximal interphalangeal joint of the right fifth toe.

**Figure 2:**
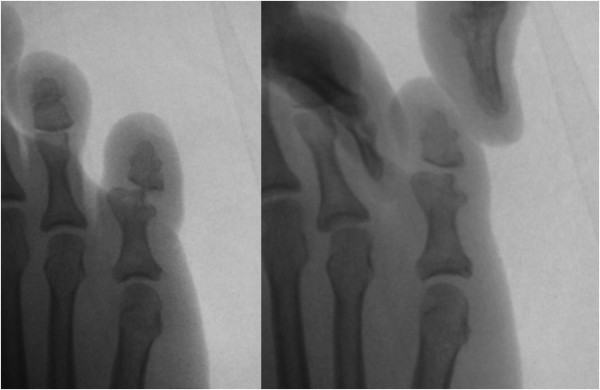
Fluoroscopic images showing the reducible but unstable of the proximal interphalangeal joint.

A 47-year-old woman in good general health, with no other co-morbidities presented with a long history of chronic right fifth toe pain for 30 years. She reported a history of injuring her right foot when playing sport for 30 years. She when landing recalled that she had twisted her right foot; moreover, the foot was not well treated at that time of the injury. The only treatment for the fifth toe was with the temporary bandage to reduce the deformity. Then, the PIP joint of the fifth toe was redislocated for several times in 6 months after the first injury and the same treatment was attempted each time. After that, the fifth toe gradually became permanently swollen and painful on movement. When the patient presented in the out patient clinic with chronic right fifth toe pain and swelling, (Figure 1), the toe was displaced in the dorsolateral plane. The plain radiograph showed a simple dislocation of PIP joint in the dorsolateral direction. The articular cartilage of middle phalanx impinged on the lateral condyle of proximal phalanx. There was no indication of definite joint destruction, erosion or unhealed fracture (Figure 2). The distal segment of the toes was unstable because of the chronic and persistent joint subluxation.

**Figure 3:**
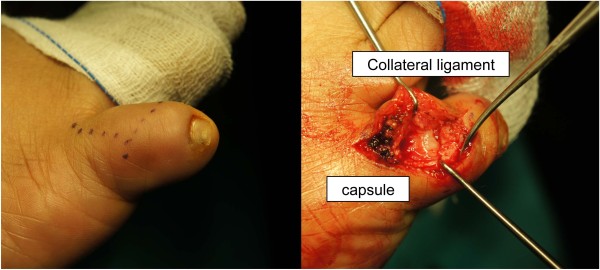
The dorsal approach showing the attenuated joint capsule and collateral ligament.

**Figure 4:**
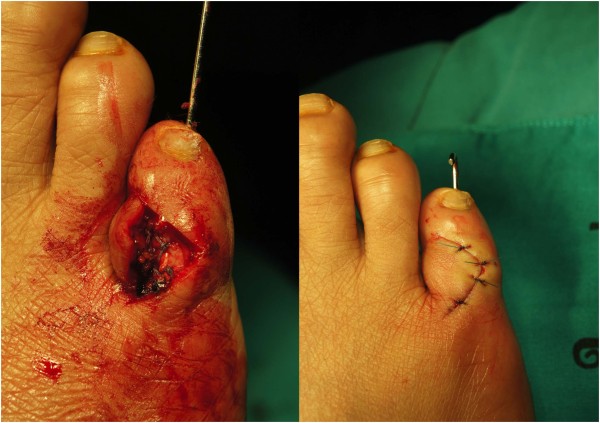
Repairing the capsular and collateral ligament and inserting the Kirschner wire creating the temporary pin fixation.

The decision was made to attempt the correction of the chronic dislocation by open reduction, as the problem had been present for many years and all previous attempts at closed reduction had been non-successful. The dislocated PIP joint was exposed via a dorsal incision, revealing that the joint capsule and attenuated lateral collateral ligament were interposed within the PIP joint space, and the articular cartilage at lateral condyle of proximal phalanx of the fifth toe became eroded (Figure 3). The joint capsule and the lateral collateral ligament were dissected away from the joint space. The tight plantar plate was released from plantar lateral aspect of the joint and the corrected position of the PIP joint was maintained by inserting a longitudinal Kirschner wire. The lateral collateral ligament was repaired anatomically. The tightened joint capsule, and the completed skin closure were done, thereby permitting soft tissue correction of the deformity (Figure 4). The sutures were removed in 2 weeks after surgery and the PIP joint correction was kept up by the retention of the in-situ Kirschner wire for 4 weeks. The wound was completely healed. The patient was able to resume normal footwear within 2 months after surgery.

**Figure 5:**
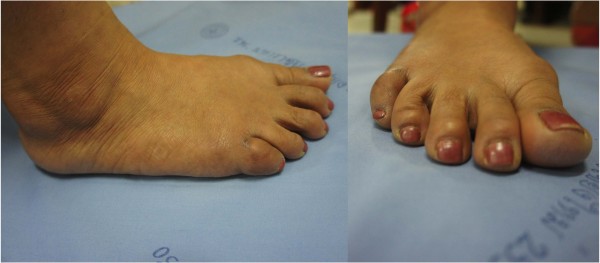
Right fifth toe, in the 12-month post-surgery.

**Figure 6:**
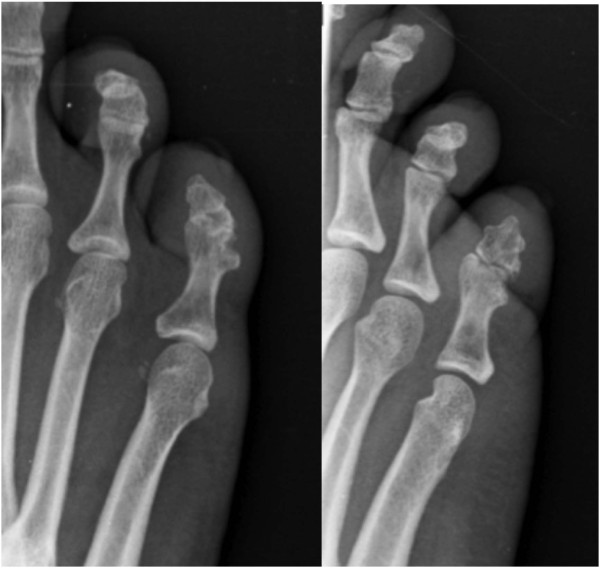
Right fifth toe: Radiograph 12-month post-surgery, showing the joint congruity and no evidence of arthritic changes.

For the 12-month post-surgery, the PIP joint of the fifth toe remained stable, painless and aesthetically pleasing (Figure 5). The patient was able to normally walk, and the range of motion of the PIP joint was within normal limits. Plain radiographs confirmed joint congruity and no signs of arthritic deterioration (Figure 6).

### Discussion

Dislocation of the PIP joint of the toe is an uncommon injury of the forefoot. The mechanism of injury usually occurs from hyperdorsiflexion or sudden application of a dorsolateral force causing traumatic abduction of the PIP joint of the toe. This is a common injury to both the great and lesser toes [1-7,9,10]. The dislocation causes instablility of the PIP joint because the soft tissues that normally ensure joint stability and congruency such as medial and lateral collateral ligaments, the fibrocartilagenous plantar plate (usually from the proximal attachment), the joint capsule and digital long and short toe extensor and flexor tendons are disrupted, compromised and injured during the forceful joint dislocation. Traumatic dislocation of the PIP joint of a toe may be successfully treated with closed reduction and adhesive neighbor strapping to adjacent toes [8]. The complex dislocation as described in this case, involving a chronic irreducible joint subluxation, or a reducible but unstable dislocation requires the surgical intervention to correct the disrupted soft tissues stabilizers, such as the medial collateral ligament, the joint capsule or the plantar plate in order to achieve joint stability.

Closed reduction is usually successful in correction of IP joint dislocation in hallucal or lesser toes. The digital deformity is initially accentuated. The longitudinal traction, allowing the joint to resume this deformity was applied and then released, so it corrected the anatomical relationship [8]. Stientra and Denner described a modification of this technique, where the dislocated joint was dorsiflexed to exaggerate the deformity. The dorsiflexion was maintained while the affected IP joint was further dorsiflexed and then plantarflexed to relocate in the anatomical joint position, usually with an audible ‘pop’ [8]. All cases should be treated initially by closed reduction, but the closed maneuver failed to achieve joint correction. The open reduction was another treatment choice [1,2,7,8]. In the case reported, only correction by closed reduction did not achieve a stable joint due to the displacement of the joint capsule, the laxity of the collateral ligament, and in part by the articular erosion of the lateral condyle of the proximal phalanx. Yang reported that approximately 50% of patients could be successfully treated by closed reduction [6].

Open reduction is best accomplished through a dorsal approach [1-7]. Temporary pin fixation is only indicated when the reduced joint is very unstable. Ultimate joint stability is improved when structures causing the buttonhole effect (i.e., the injury involving with the long extensor tendon, the distal end of the phalanx becoming “buttonholed” between the outer bands of the tendon) are removed. If the capsule, collateral ligaments, tendons or plantar plate are injured or attenuated, they should be repaired to achieve a neutral and anatomical position [1,2,6,7,9]. In our case, open reduction was performed via dorsal approach after failure of closed method. The attenuated lateral collateral ligament found to be entrapped within the joint was released. This finding was different from the previous publication in that medial collateral ligament and flexor digitorum longus tendon became entrapped in the joint. The plantar plate that contracted on the plantar lateral aspect of the joint from prolong period of subluxation and dislocation was one of the important structures causing the irreducible or reducible instability of the joint. It was released to restore joint stability. The dorsal capsule and lateral collateral ligament were repaired to achieve the stability. Temporary pin fixation was performed to enhance the joint stability during the surrounding tissue healing promoted. The pin was removed after 4 weeks to achieve stability and painless PIP joint. Radiographic examination following closed reduction is strongly recommended [8], as chronic irreducible dislocation of the lesser toes could result from the lack of definitive radiographic evaluation since closed reduction with inadequate immobilization was insufficient.

## Conclusions

Traumatic dislocation of the interphalangeal joint of the fifth toe should be diagnosed at the earliest stage which was treated initially by adequate tractional reduction of the joint incongruency with relating to soft splintage immobilization in order to prevent subsequent complex chronic joint subluxation. In cases where this approach is not successful or pain persists open reduction, reposition and restabilization of the dislocated joint and its associated soft tissue structures are indicated to achieve a functional toe.

## Consent

Written informed consent was obtained from the patient for the publication of this report and any accompanying images.

## Competing interests

The authors declare that they have no competing interests.

## Authors’ contributions

All authors read and approved the final manuscript.
